# Biochemical characterization of a recombinant acid phosphatase from *Acinetobacter baumannii*

**DOI:** 10.1371/journal.pone.0252377

**Published:** 2021-06-02

**Authors:** Elizabeth Smiley-Moreno, Douglas Smith, Jieh-Juen Yu, Phuong Cao, Bernard P. Arulanandam, James P. Chambers

**Affiliations:** 1 South Texas Center for Emerging Infectious Disease and Department of Biology, The University of Texas at San Antonio, San Antonio, Texas, United States of America; 2 School of Medicine, University College Dublin, Dublin, Ireland; Weizmann Institute of Science, ISRAEL

## Abstract

Genomic sequence analysis of *Acinetobacter baumannii* revealed the presence of a putative Acid Phosphatase (AcpA; EC 3.1.3.2). A plasmid construct was made, and recombinant protein (rAcpA) was expressed in *E*. *coli*. PAGE analysis (carried out under denaturing/reducing conditions) of nickel-affinity purified protein revealed the presence of a near-homogeneous band of approximately 37 kDa. The identity of the 37 kDa species was verified as rAcpA by proteomic analysis with a molecular mass of 34.6 kDa from the deduced sequence. The dependence of substrate hydrolysis on pH was broad with an optimum observed at 6.0. Kinetic analysis revealed relatively high affinity for PNPP (K_m_ = 90 μM) with V_*max*_, *k*_cat,_ and *K*_cat_/*K*_m_ values of 19.2 pmoles s^-1^, 4.80 s^-1^(calculated on the basis of 37 kDa), and 5.30 x 10^4^ M^-1^s^-1^, respectively. Sensitivity to a variety of reagents, *i*.*e*., detergents, reducing, and chelating agents as well as classic acid phosphatase inhibitors was examined in addition to assessment of hydrolysis of a number of phosphorylated compounds. Removal of phosphate from different phosphorylated compounds is supportive of broad, *i*.*e*., *‘*nonspecific’ substrate specificity; although, the enzyme appears to prefer phosphotyrosine and/or peptides containing phosphotyrosine in comparison to serine and threonine. Examination of the primary sequence indicated the absence of signature sequences characteristic of Type A, B, and C nonspecific bacterial acid phosphatases.

## Introduction

Phosphatases (EC 3.1.3) are a diverse, ubiquitous group of enzymes that hydrolyze phosphoesters from a wide variety of compounds [1]. These enzymes have been broadly grouped according to their pH optimum, *i*.*e*., acid, neutral or alkaline. Acid phosphatases have been further grouped according to molecular weight, substrate specificity, response to inhibitors, physical appearance, and sequence homology into two large families, *i*.*e*., specific and nonspecific. Both are widely distributed among Gram-positive and Gram-negative bacteria. Some are released in soluble form, *i*.*e*., secreted, or retained in the periplasmic space either free or membrane bound [2, 3]. ‘Specific’ phosphatases have been shown to play a role in cellular processes removing phosphate, the most common metabolomic functional group [1, 4]; whereas, ‘nonspecific’ phosphatases (NSAPs) are considered physiologically important in both utilization of phosphoesters, and in gene expression [5]. NSAPs have been further grouped into three classes, *i*.*e*., A, B, and C on the basis of physico-chemical properties, and presence of key amino acid signature sequences [6–8].

*Acinetobacter baumannii* is an opportunistic pathogen which has become a medically relevant nosocomial pathogen accounting for approximately 2% of all healthcare associated infections in the United States [9]. The rate of multidrug resistant phenotypes associated with *Acinetobacter* infections has merited *Acinetobacter* as being considered a severe threat requiring use of last line treatment options, *i*.*e*., use of carbapenenems, tigecycline, and colistin therapeutic agents [10]. Epidemiological data indicate *Acinetobacter spp* to have acquired multi-drug resistant phenotypes faster than almost all other Gram-negative bacteria [11].

Genomic sequence data suggest the presence of a putative acid phosphatase (NCBI Reference Sequence WP_000749225.1). Given that acid phosphatases (EC 3.1.3.2) have been implicated as intracellular pathogen virulence factors [12, 13] and the emergence of this pathogen, we report here partial characterization of an *Acinetobacter baumannii* recombinant acid phosphatase (rAcpA). The role of this enzyme in *Acinetobacter baumannii* pathogenesis is unknown and the focus of current efforts.

## Materials and methods

### Cloning and protein expression

The complete genome of *Acetinobacter baumannii* (ATCC 17978 strain) has been sequenced and annotated [14]. An *Acinetobacter baumannii* acid phosphatase gene (NCBI Accession CP000521, region: 2058753–2059719, encoding 322 amino acids) construct was synthesized with a C-terminal histidine tag, and inserted into a pET23a(+) expression vector via *Nde*I/*Hin*dIII restriction sites (Genscript, *Inc*.). The resulting pET23a-acid phosphatase (AcpA) bearing plasmid was used to transform *Escherichia coli* Rosetta by electroporation using a BioRad Gene Pulser Xcell apparatus. Bacterial transformants harboring pET23a-AcpA were selected on LB agar plates containing ampicillin (100 μg/ml) and chloramphenicol (50 μg/ml) followed by PCR confirmation using a pair of AcpA gene specific primers. For expression of rAcpA protein, an overnight culture of pET23a-AcpA transformant was used to inoculate 1 L LB broth while shaking at 225 RPM at 37 ^0^C to an OD_600_ of 0.6. Isopropyl β-D-1-thiogalactoside (IPTG, Sigma Chemical Co.) was then added (1 mM final concentration), incubated for an additional 4 hours after which time bacterial cells were pelleted at 4000 x g for 10 minutes at 5 ^0^C. Following decanting of supernatant, the pellet was stored at -20 ^0^C.

### Preparation of recombinant lysate

Pellet material was resuspended in 20 mL ice-cold lysis buffer [50 mM NaH_2_PO_4_ buffer, pH 8 containing 300 mM NaCl, 10 mM imidazole, and 2 protease inhibitor cocktail tablets (Sigma Chemical Co.)]. The pellet suspension was sonicated using a Misonix Ultrasonic Liquid Processor (XL-200 Series) at an output wattage of 10 while partially immersed in an ice slurry. Sonicated lysate was centrifuged at 6000 x g for 15 minutes at 5 ^0^C. Supernatant was decanted and 20 mL lysis buffer containing 8 M urea was added to the pellet, sonicated a second time, and allowed to stand in an ice slurry for one hour to extract and free rAcpA from bacterial inclusion bodies. Recombinant protein containing supernatant was obtained by centrifugation at 6000 x g for 15 minutes, and His-tagged rAcpA was subjected to affinity chromatography purification using Ni^2+^-nitrilotriacetic acid (Ni-NTA) Agarose beads (Qiagen).

### Nickel column purification

Ni-NTA Agarose beads (3 mL) were washed (3 times) with 2 mL cold lysis buffer and pelleted at 200 x g for 2 minutes. Recombinant protein was added to the washed beads, mixed thoroughly overnight at 5 ^0^C using an inverting rotator, transferred to a small glass column, and allowed to settle (final bed volume dimensions = 1.7 x 13 cm). The column was first washed with 25 mL lysis buffer containing 8M urea followed by 20 mL cold wash buffer (50 mM NaH_2_PO_4_ buffer, pH 8 containing 300 mM NaCl, 40 mM imidazole, and 8 M urea). Bead-bound recombinant protein was eluted by sequential addition of ten 1 mL applications of cold elution buffer (50 mM NaH_2_PO_4_ buffer, pH 8 containing 300 mM NaCl, 250 mM imidazole, and 8 M urea). Eluates (1 mL) were collected in microcentrifuge tubes partially immersed in an ice slurry.

### Removal of urea

Eluted *Acinetobacter baumannii* rAcpA was subjected to dialysis using a 500–1000 molecular weight cutoff dialysis membrane (Fisher Scientific). Recombinant protein eluates were pooled (~ 5–7 mL), and dialyzed sequentially against 200 mL dialysis buffer (0.050 M sodium acetate buffer, pH 6.5) containing 4, 2, 1, and 0 M urea. Respective dialysates were allowed to equilibrate at 5 ^0^C for two hours. The final dialysis against buffer containing no urea, *i*.*e*., 0 M was carried out twice. Dialysate was removed and aliquoted (1 mL) to which 50 μL glycerol was added followed by storage at -20 ^0^C. This served as source of purified recombinant protein used in this study.

### Acid phosphatase assay

Unless indicated otherwise, reaction mixtures contained 2.0 mM PNPP (Sigma Chemical Co.) or phosphorylated compounds (Sigma Chemical Co.), 0.20 M MES (Sigma Chemical Co.) buffer, pH 6.0, 2.0 mM NiCl_2_, and 0.080–0.30 μg total *Acinetobacter baumannii* rAcpA protein, and were brought to a final concentration of 0.186 μg/μL protein with addition of bovine serum albumin (BSA, BioRad Laboratories) in a total reaction volume of 200 μL (determination of released phosphate) or 300 μL (determination of released paranitrophenol). All incubations were carried out for 30 minutes at 37 ^0^C after which time reactions were placed in an ice slurry for 3 minutes followed by addition of enzyme to the respective blanks and reactions were terminated by heating at 65 ^0^C for 10 minutes followed by immersion in an ice slurry for 3 minutes. Released phosphate was determined by addition of 1.0 mL BIOMOL GREEN phosphate reagent (Enzo Life Sciences), and monitored at 620 nm using a Genesys 10 UV Scanning Spectrophotometer (Thermo Scientific). Paranitrophenol was monitored at 405 nm following addition of 1.7 mL 0.5 M Glycine buffer, pH 10. Following subtraction of blank values, paranitrophenol and released phosphate were quantitated using paranitrophenol and phosphate standard curves, respectively. All reactions, *i*.*e*., generation of paranitrophenol and release of free phosphate were linear with both time (30 minutes) and assay protein (0.30 μg, ~ 13.2 nM). Specific activity is expressed as nmoles paranitrophenol or free phosphate liberated mg^-1^s^-1^.

### Protein determination

Protein was determined using the Bradford dye-binding procedure [15] per manufacturer’s recommendation (BioRad Laboratories) with BSA as protein standard.

### PAGE analysis

Recombinant protein (~0.5–1.0 μg) and pre-stained standard ladder were added to 10 μL 2x Laemmli sample buffer under reducing conditions (2.0% v/v β-mercaptoethanol), and denatured at 95 ^0^C for 2 minutes. Respective samples were applied onto 4–20% precast gradient polyacrylamide gels partitioned in 1X Tris Glycine buffer containing SDS. Electrophoresis was carried out at constant voltage (80 volts) for 90 minutes at room temperature using a Power Pac 3000 power supply, and Mini Protein II electrophoretic apparatus. Protein was stained using Coomassie Brilliant Blue R-250. All electrophoresis reagents (sample buffers, running buffers, standards, precast gels, and gel stain) and equipment were obtained from Bio-Rad Laboratories.

### Proteomic analysis

The predominant Coomassie Blue stained protein band (~37 kDa) resolved by electrophoresis of nickel-affinity purified protein (*cf*., PAGE analysis) was excised, subjected to trypsin digestion, and analyzed by matrix-assisted laser desorption ionization-time of flight (MALDI-TDF) mass spectrometry in the Proteomics and Biomarkers Core of the University of Texas at San Antonio.

## Results

### Expression and purification of rAcpA

**Fig 1:**
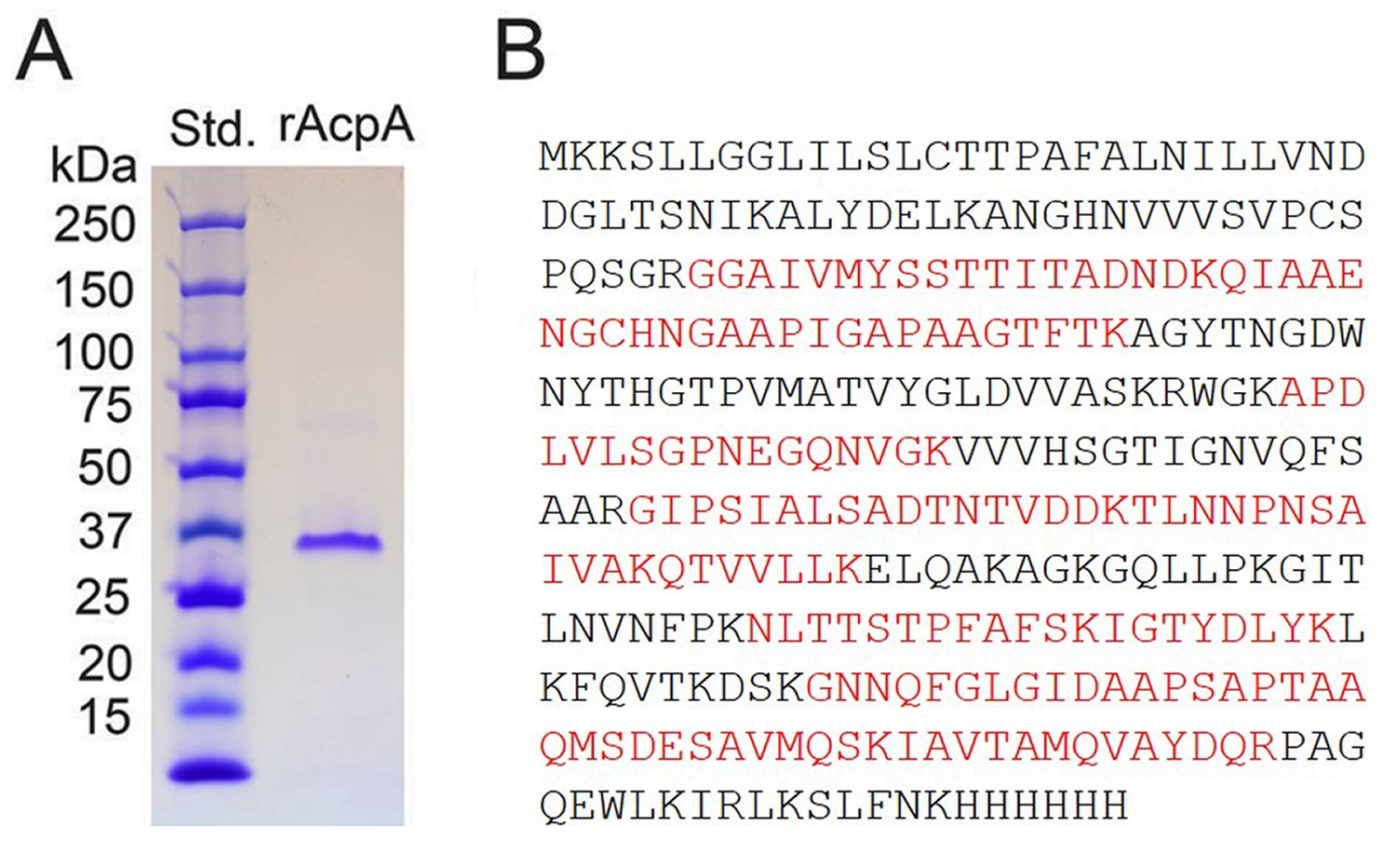
Purification and proteomic analysis of *Acinetobacter baumannii* rAcpA. (A) SDS-PAGE analysis of nickel-affinity purified rAcpA. (B) Proteomic analysis. The 9 matching peptide sequences are shown in red.

A putative *Acinetobacter baumannii* acid phosphatase (AcpA) was expressed as a His(6x)-tagged recombinant protein in *E*. *coli*. Recombinant protein was purified to near homogeneity by nickel affinity column chromatography and migrated as a species of ~37 kDa under denaturing/reducing conditions (Fig 1A) in agreement with the molecular weight of 34.6 kDa from the deduced protein sequence. Proteomic analysis of purified rAcpA revealed 9 peptides (sequences in red) that coincided with that of the deduced *Acinetobacter baumannii* rAcpA with a 49% overall protein coverage (Fig 1B).

### Dependence of rAcpA activity on pH and divalent cations

**Fig 2:**
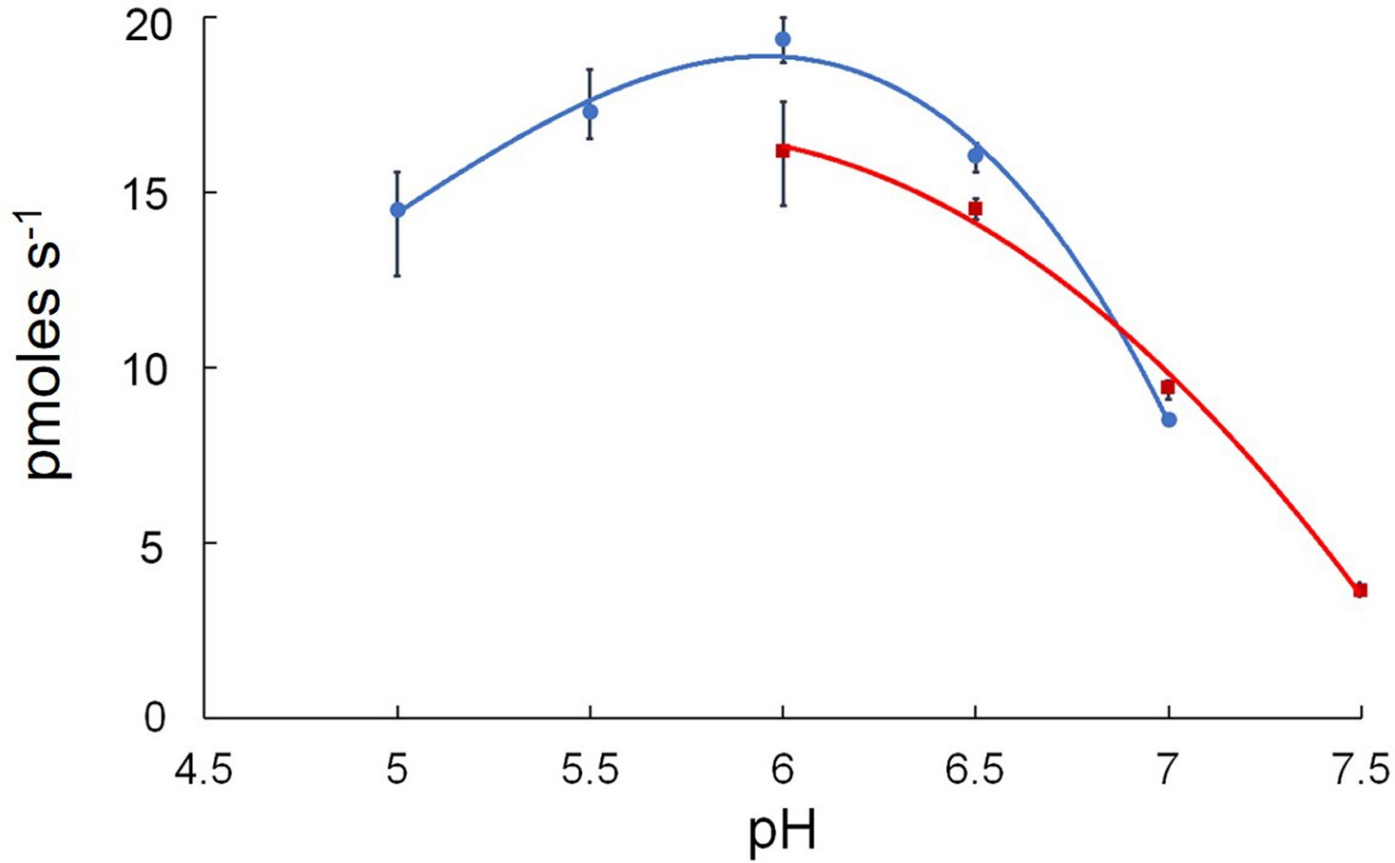
Determination of optimal pH. *Acinetobacter baumannii* rAcpA activity (pmoles s^-1^) was determined as a function of pH using 0.2 M HEPES (solid circles, blue line) and 0.2 M MES (solid squares, red line) buffered reaction mixtures. Error bars represent 1 standard deviation from the mean.

**Fig 3:**
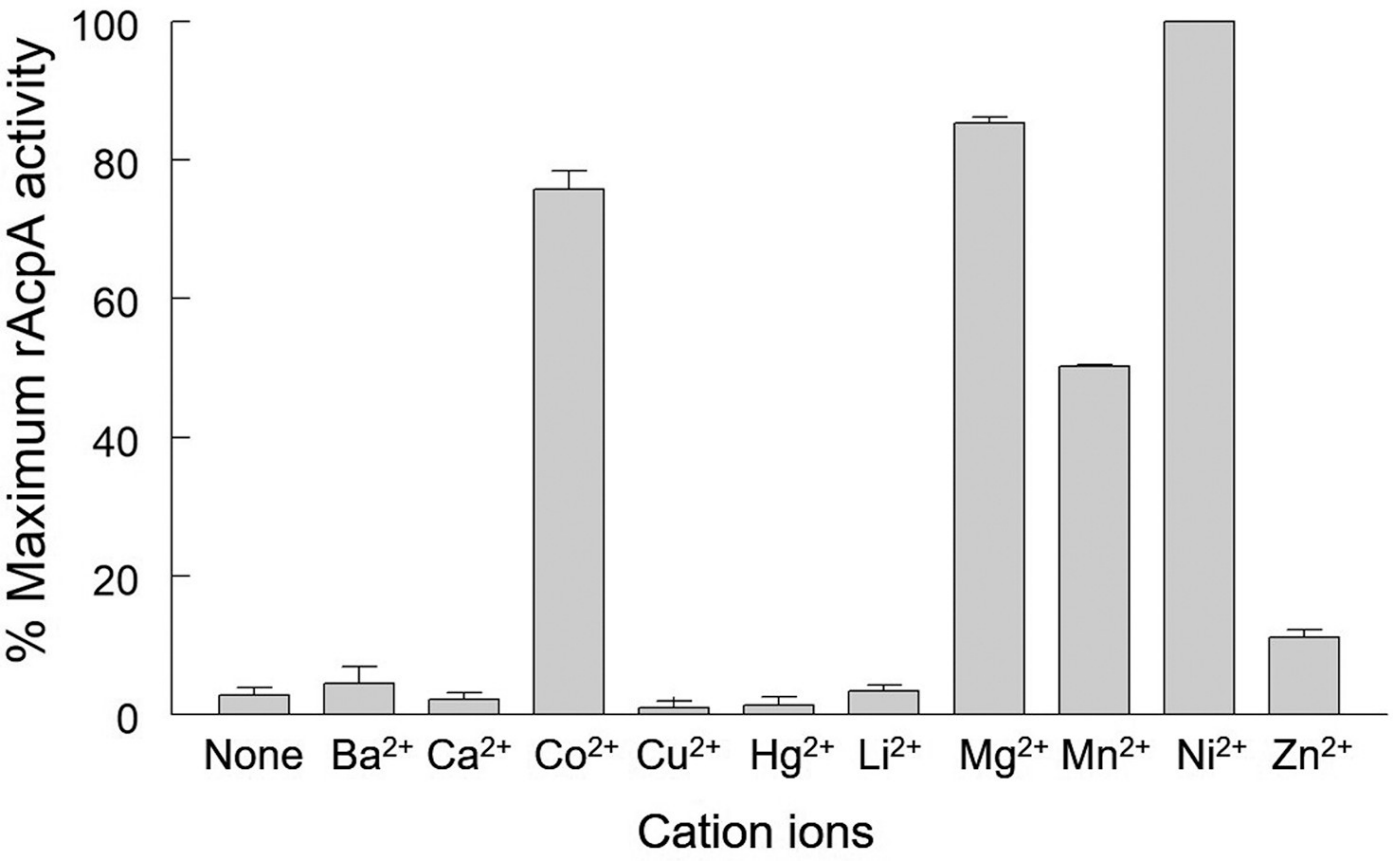
Effect of divalent cations on *Acinetobacter baumannii* rAcpA activity. Reactions were carried out at pH 6.0 in the presence of indicated divalent cations (2 mM final concentration). Activity is expressed as percent of rAcpA activity (pmoles s^-1^) observed in the presence of NiCl_2_. Error bars represent 1 standard deviation from the mean.

The dependence of rAcpA PNPP hydrolysis on pH was carried out in the presence of 2.0 mM NiCl_2_ buffered with 0.2 M HEPES (pH 5.0–7.0) or MES (pH 6.0–7.5). As shown in Fig 2, rAcpA exhibited maximum hydrolysis of PNPP at pH 6.0. Shown in Fig 3 is the dependence of PNPP hydrolysis by rAcpA on various cations at pH 6.0. Maximum hydrolysis was observed in the presence of 2.0 mM NiCl_2_ with MgCl_2,_ CoCl_2_, MnCl_2,_ and ZnCl_2_ exhibiting 85, 76, 50, and 11% that observed for NiCl_2_, respectively.

### Effect of inhibitors, detergents, and reducing agents

Shown in Table 1 is the effect of common phosphatase inhibitors, detergents, and reducing agents on rAcpA PNPP hydrolysis. Inhibition, *i*.*e*., 50% (IC_50_) by sodium orthovanadate and molybdate was 125 and 350 μM, respectively. EDTA, sodium tartrate, and sodium pyrophosphate were moderately inhibitory (IC_50_ = 1.25, 2.0, and 2.5 mM, respectively) while sodium fluoride, and sodium phosphate inhibited 50% at 6.0, and 9.0 mM, respectively. In contrast to Triton X-100 which inhibited 50% at 5.1 mM (0.32% v/v), the nonionic detergent Tween 20 stimulated ~1.4-fold at 3.2 mM (0.40% v/v). Although anionic detergents deoxycholate and taurocholate exhibited millimolar IC_50_ values, *i*.*e*., 1.5 and 4.0, respectively, dodecyl sulfate was very inhibitory (IC_50_ = 100 μM). *Acinetobacter baumannii* rAcpA exhibited some sensitivity albeit low (~19%) to cysteamine phosphate, a classical inhibitor of alkaline phosphatase. Although moderately sensitive to β-mercaptoethanol (IC_50_ = 10 mM), hydrolysis of PNPP was considerably more sensitive to dithiothreitol and dithioerythritol (IC_50_ = 3.0 and 2.0 mM, respectively). The enzyme was observed to be insensitive to okadaic acid.

**Table 1:** Effect of common inhibitors, detergents, and reducing agents on *Acinetobacter baumannii* rAcpA activity.

Compounds	IC_50_ (mM)^a^	Max. Conc. Tested (mM)	Percent Control^d^
Sodium Pyrophosphate	2.5	10	
Sodium Tartrate	2.00*	10	
Sodium Phosphate	9	20	
Sodium Fluoride	6	20	
Molybdate	0.35	2	
Vanadate	0.125	2	
EDTA	1.25	5	
Sodium Taurocholate	4.00*	10	
Sodium Deoxycholate	1.5	10	
Triton X100	5.10^b^*	16	
Tween 20^c^	N/A	3.2	140
Sodium Dodecyl Sulfate	0.10	1.0	
Cysteamine S-Phosphate	ND	2.0	81
β-Mercaptoethanol	10.0	25	
Dithiothreitol	3.0	10	
Dithioerythritol	2.0	10	
Okadaic acid	ND	0.0001	100

^a^ Inhibitor concentration rendering 50% inhibition (IC_50_) was determined by extrapolation from the 50% inhibition value. Each compound tested was dissolved in dH_2_O and analyzed out to the indicated maximum concentration (5 replicates for each concentration tested and corresponding blank).

^b^ 0.32% corresponds to 5.10 mM. Molarity-calculated on 625 g/mole.

^c^ 0.40% corresponds to 3.2 mM. Molarity-calculated on 1,225 g/mole.

^d^ Percent control = (Activity in presence of compound)/(Activity in absence of compound) x100.

* Apparent IC_50_-extrapolated from the linear portion of the titration curve prior to leveling off and reaching 50% activity.

ND = Not determined

N/A = Not applicable

### rAcpA enzymatic kinetics

**Fig 4:**
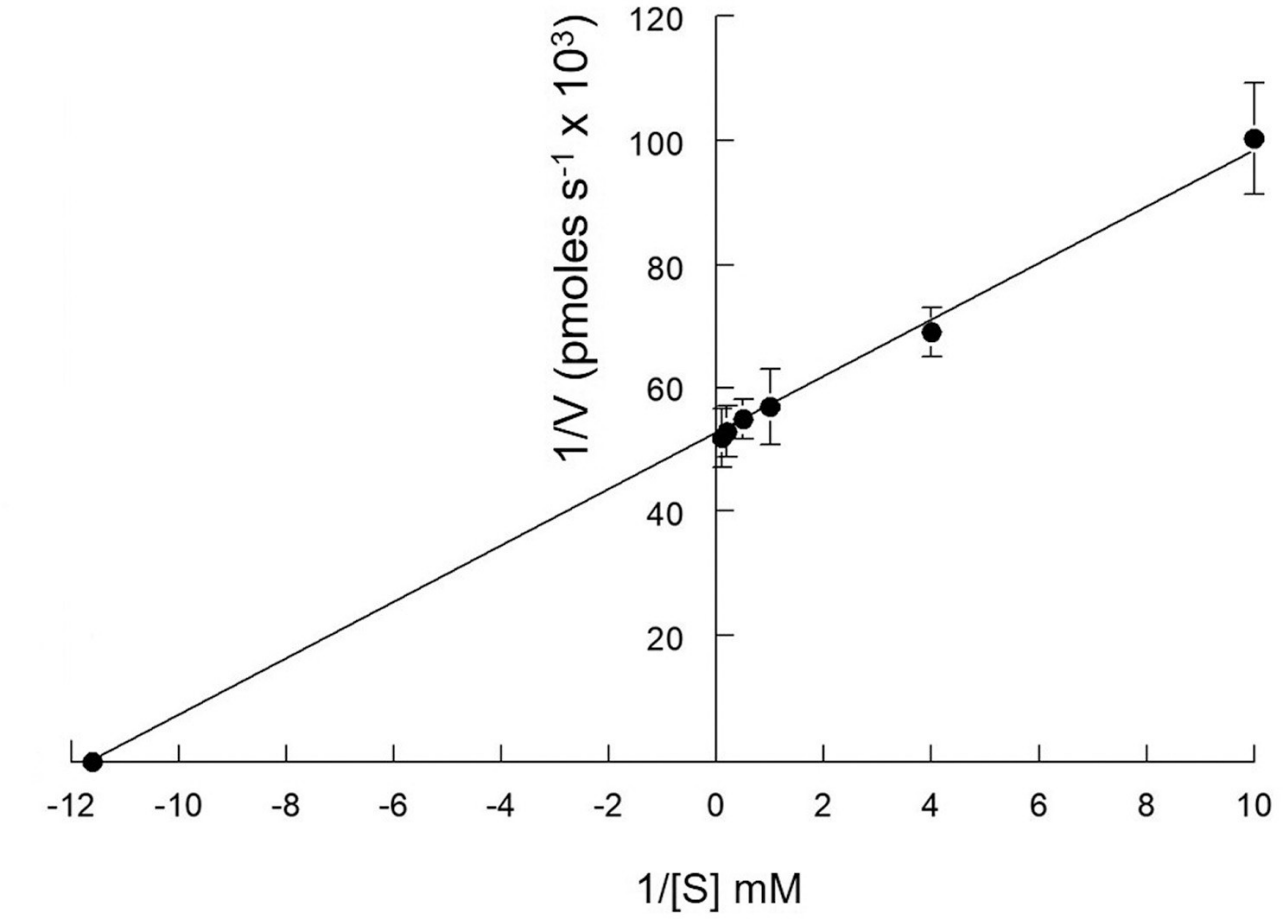
Determination of *K*_m_ and *V*_max_. Enzymatic activity (0.149 μg rAcpA) was determined over a broad range of PNPP concentrations (0.10–10.0 mM) in replicates of five for each substrate concentration tested and corresponding blank. *K*_m_ and *V*_max_ values were derived from standard Lineweaver-Burk plot analysis. Error bars represent 1 standard deviation from the mean.

The effect of increasing PNPP substrate on *Acinetobacter baumannii* rAcpA activity was determined. Shown in Fig 4 is a Lineweaver-Burk plot from which K_m_ and V_*max*_ values of 90 μM and 19.2 pmoles s^-1^, respectively, were derived. The turnover number (k_*cat*_) and catalytic efficiency (k_*cat*_/K_*m*_) were calculated to be 4.80 s^-1^ and 5.30 x 10^4^ M^-1^s^-1^, respectively.

### rAcpA hydrolyzes different phosphorylated substrates

*Acinetobacter baumannii* rAcpA was observed to remove phosphate from an array of substrates including nucleotides, sugars, metabolites, vitamin derivatives, phosphorylated amino acids, and phosphorylated peptides (Table 2).

**Table 2:** Hydrolysis of various phosphorylated compounds by *Acinetobacter baumannii* rAcpA.

Compound^a^	Relative Activity^b^	Compound^a^	Relative Activity^b^
Pyridoxal Phosphate	35.3	3’-AMP^c^	63.0
Thiamine Pyrophosphate	3.6	AMP	27.0
Thiamine Monophosphate	12.3	ADP	1.9
Phospho-L-Threonine	4.5	ATP	1.1
Phospho-L-Tyrosine	31.5	CMP	4.1
Phospho-L-Serine	0.4	CDP	0.0
Phosphoethanolamine	1.8	CTP	0.0
Phosphocholine	2.0	GMP	0.0
β-Glycerol Phosphate	40.0	GDP	1.0
Ribose-5-Phosphate	20.0	GTP	0.0
Galactose-1-Phosphate	0.0	IMP	6.0
Fructose-1,6-bisphosphate	6.6	IDP	0.5
Glucose-6-Phosphate	2.7	ITP	0.0
Mannose-6-Phosphate	10.6	TMP	27.3
Trehalose-6-Phosphate	4.0	TDP	3.0
PEP	7.6	TTP	0.0
Phosphocreatinine	0.6	UMP	8.7
Phytic acid	0.0	UDP	1.8
Serine Phosphopeptide	0.0	UTP	0.0
Threonine Phosphopeptide	0.0	NADP^+^	3.7
Tyrosine Phosphopeptide	20.0	NADPH	3.5
β-Casein	1.2		

^a^ Compounds were dissolved in dH_2_O. With the exception of phosphopeptides and β-Casein, all phosphorylated compounds were tested at 2.0 mM final concentration. Phosphopeptides

R-R-A-pS-V-A, K-R-pT-I-R-R, T-S-T-E-P-Q-pY-Q-P-G-E-N-L, and β-Casein were all tested at 0.1 mM final concentration.

^b^ Relative Activity (%) = (nmoles mg^-1^s^-1^ phosphate released from phosphorylated compounds)/(nmoles mg^-1^s^-1^ paranitrophenol released from PNPP) x 100.

0% = Experimental values (5 replicates)–Blank values (5 replicates).

^c^With the exception of 3’-AMP, all nucleoside mono-, di-, and triphosphates listed are 5’.

### *Acinetobacter baumannii* AcpA exhibits high homology with the *Escherichia coli* SurE protein

**Fig 5:**
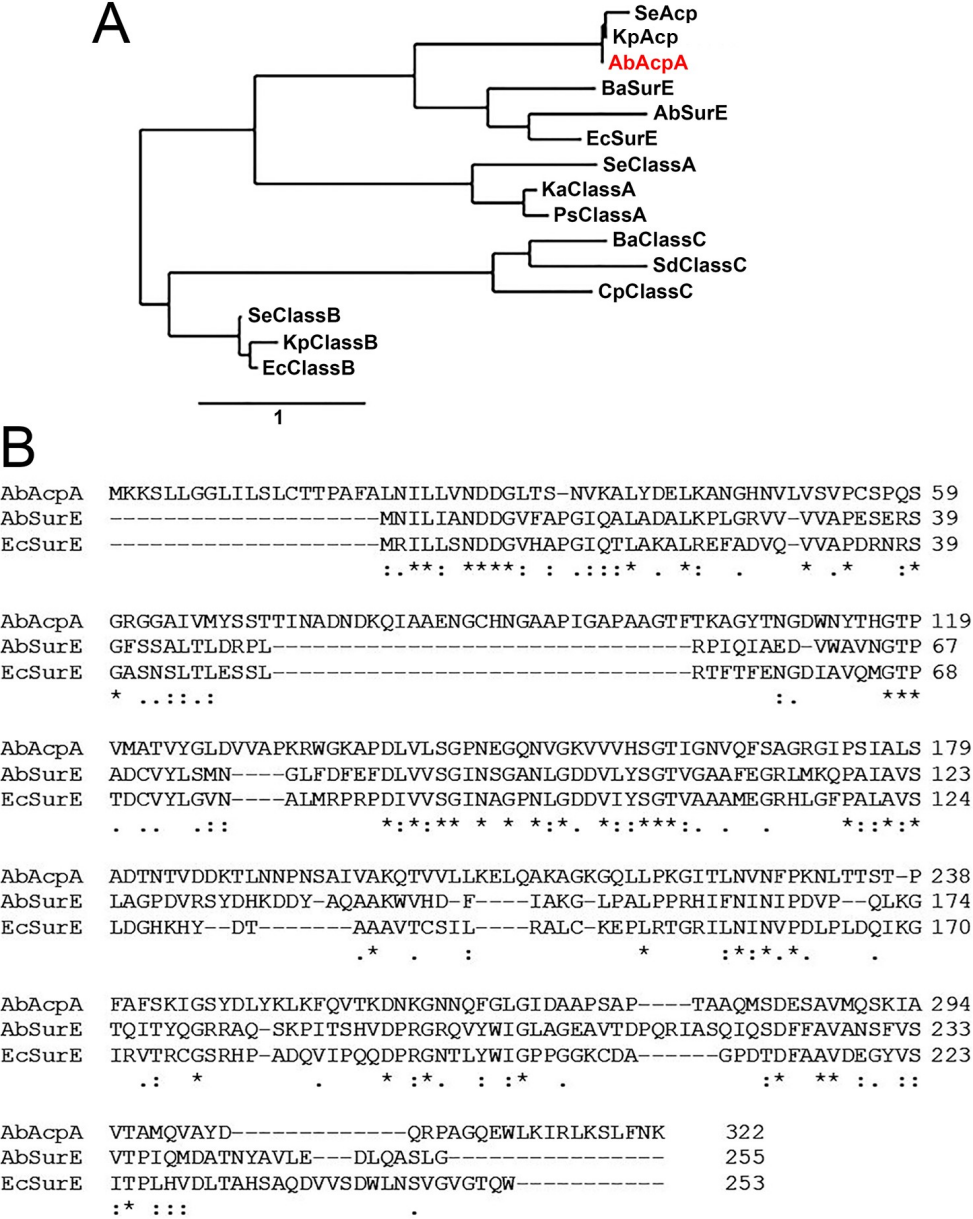
Phylogenic analysis of *Acinetobacter baumannii* AcpA. (A) Phylogenetic tree constructed using amino acid sequences of AbAcpA and other indicated bacterial phosphatases including SurE proteins and 3 classes of non-specific acid phosphatases. The names of bacteria along with the NCBI accession numbers for each protein are as follows: SeAcp (*Salmonella enterica*; ECE6726571.1), KpAcp (*Klebsiella pneumoniae*; AAL59317.1), AbAcpA (*Acinectobacter baumannii*; ABO12194.2), BaSurE (*Brucella abortus*; WP_002966786.1), AbSurE (*Acinectobacter baumannii*; A3M7F7), EcSurE (*Escherichia coli*; WP_001472109.1), SeClassA (*Salmonella enterica*; CAA41760.1), KaClassA (*Klebsiella aerogenes*; ABW37174.1), PsClassA (*Providencia stuartii*, CAA46032.1), BaClassC (*Bacillus* sp.; ABO69628.1), SdClassC (*Streptococcus dysgalactiae*; CAA73175.1), CpClassC (*Clostridium perfringens*; ACB11490.1), SeClassB (*Salmonella enterica*; AAW22868.1), KpClassB (*Klebsiella pneumonia*; SSW83847.1), and EcClassB (*Escherichia coli*; CAA60534.1). (B) Amino acid sequence alignment of AbAcpA, AbSurE, and EcSurE.

*A*. *baumannii* AcpA (AbAcpA) has been annotated as an acid phosphatase. Gandhi and Chandra classified bacterial non-specific acid phosphatases into A, B and C classes [5]. Protein BLAST (via NCBI) and phylogeny (PhyML, via http://www.phylogeny.fr; [16, 17]) analyses reveal that the AbAcpA is closely related to the well-characterized *E*. *coli* SurE protein (EcSurE; NCBI Reference Sequence: WP_001472109.1), but distant from other non-specific acid phosphatases as shown in Fig 5A. The EcSurE protein is a metal ion-dependent phosphatase [18] that dephosphorylates various ribo- and deoxyribonucleoside 5′-monophosphates and ribonucleoside 3′-monophosphates, and plays an important role in bacterial stationary phase survival [19]. *Acinetobacter baumannii* also expresses a SurE-like protein (AbSurE; GenBank A3M7F7) with 69.2% amino acid similarity (43.6% identity) to EcSurE, and 47.3% amino acid similarity (21.9% identity) to AbAcpA. Shown in Fig 5B is a multiple sequence alignment of AbAcpA, AbSurE, and EcSurE proteins using Clustal W [20, 21]. The presence of a unique leader sequence in AbAcpA (Fig 5B) suggests this protein may be secreted while AbSurE resides primarily inside the bacterium.

## Discussion

C-terminus His(6x)-tagged *Acinetobacter baumannii* rAcpA migrated as a monomer of ~37 kDa under reducing denaturing conditions which is in good agreement with the predicted molecular weight from protein sequence (Fig 1B), *i*.*e*., 34,619. The identity, *i*.*e*., sequence of the recombinant protein synthesized from the gene construct was verified by proteomic analysis. Examination of the primary sequence revealed the absence of GSYPSGHT, FDIDDTVLFSSP, and bipartite sequence motifs [IV]-[VAL]-D-[IL]-D-E-T-[VM]-L-X-[NT]-X(2)-Y and [IV]-[LM]-X(2)-G-D-[NT]-L-X-D-F signature sequences of Class A, B, and C nonspecific acid phosphatase, respectively [3, 22]. Additionally, this phosphatase shares no other sequences unique to Class A, B, and C bacterial phosphatases as shown by Gandhi and Chandra [5]. Although the bipartite Class C signature sequence with defining aspartic acid residues is missing in *Acinetobacter baumannii* rAcpA, the primary sequence does contain multiple aspartic acid (D) residues.

Maximum hydrolysis of PNPP by *Acinetobacter baumannii* rAcpA was observed at pH 6.0 in the presence of 2.0 mM NiCl_2_. Kinetic analysis revealed micromolar affinity for PNPP (K_*m*_ = 90 μM), turnover number of 4.80 s^-1^, and a catalytic efficiency of 5.30 x 10^4^ M^-1^s^-1^. *Acinetobacter baumannii* rAcpA is sensitive to early transition metal oxyanions, *i*.*e*., vanadate and molybdate (μmolar IC_50_ values) compared to that observed for other compounds tested (mM IC_50_ values). Sensitivity to vanadate is suggestive of involvement of transiently phosphorylated histidine residues during cleavage of the phosphomonoester O-P bond [23, 24]. The classic inhibitor of alkaline phosphatase, *i*.*e*., cysteamine phosphate had no strong effect although a small degree, *i*.*e*., ~19% inhibition was observed. Inhibition by EDTA (IC_50_ = 1.25 mM) is consistent with a divalent metal cation requirement for maximum activity. Pyrophosphate was shown to reduce activity by 50% at 2.5 mM. Consistent with the presence of 3 cysteine residues accommodating formation of one disulfide bridge, the effect of reducing agents suggest differential accessibility with 10 mM 2-mercaptoethanol reducing activity by 50% in contrast to 3.0 and 2.0 mM dithiothreitol (*trans* isomer) and dithioerythritol (*cis* isomer), respectively. The monofunctional sulfhydral group reagent mercury was very inhibitory (*cf*., Fig 3). This enzyme appears more sensitive to tartrate (IC_50_ = 2.0 mM) but only moderately sensitive to fluoride (IC_50_ = 6.0 mM), classical and common inhibitors of high molecular weight acid phosphatases [13]. Okadaic acid, did not inhibit *Acinetobacter baumannii* rAcpA at 100 nM. Although 5 times the IC_50_ value observed for inhibition of serine/threonine phosphatase protein 1, this is consistent with *Acinetobacter baumannii* rAcpA not being a member of this family of phosphatases [25, 26].

A comparison of hydrolysis of phosphoester bearing molecules to that of PNPP by *Acinetobacter baumannii* rAcpA revealed several phosphorylated compounds as potential substrates. Most notable was pyridoxal phosphate (35.3%), ribose-5-phosphate (20.0%), 3’-AMP (63.0%), 5’-AMP (27.0%), TMP (27.3%), and thiamine monophosphate (12.3%). Some of the phosphorylated compounds evaluated were not hydrolyzed relative to PNPP, *i*.*e*., galactose-1-phosphate, phytic acid, CDP, CTP, GMP, GTP, ITP, TTP, and UTP. Nucleotides ADP, ATP, GDP, IDP, and UDP were marginally hydrolyzed, *i*.*e*., < 2% that of PNPP; whereas, CMP, IMP, TDP, and UMP exhibited higher relative hydrolysis values from 3.0–8.7%.

Reversible phosphorylation is an important means of regulation in prokaryotes [27]. Numerous serine/threonine/tyrosine phosphorylated proteins have been observed in bacteria [28–30]. Likewise, serine/threonine kinases from phylogenetically diverse bacteria have been described [30]. Bacterial phosphoprotein-metal dependent phosphatases have been shown to mediate dephosphorylation of phosphoserine or phosphothreonine residues, and a 2C-like phosphoprotein phosphatase has been described in *E*. *coli* [31]. However, these phosphatases have been shown to hydrolyze PNPP only in the presence of Mn^2+^ and are insensitive to vanadate properties inconsistent with the *Acinetobacter baumannii* rAcpA. Although the phosphatase from *Acinetobacter baumannii* removed phosphate from the phosphorylated amino acid phospho-L-tyrosine (31.5% that of PNPP, assayed at 2.0 mM) and tyrosine phosphopeptide (20% that of PNPP but assayed at 100 μM, ~ 1 *K*_m_ value), little to no phosphate was released from phospho-L-threonine (4.5%) and threonine phosphopeptide (0%), respectively. β-casein, a 30 kDa protein containing 5 serine residues exhibited 1.2% hydrolysis relative to PNPP while 0.4, and 0% hydrolysis was observed for phospho-L-serine and serine phosphopeptide, respectively.

The removal of phosphate from ribose-5-P, AMP, thiamine monophosphate, and pyridoxal phosphate (vitamin B_6_) may constitute a means of interdiction of host response to the organism. Alternatively, removal of phosphate from 3’ and 5’ nucleoside monophosphate, *i*.*e*., AMP, and 5’ nucleoside monophosphates TMP, CMP, IMP, TDP, and UMP is consistent with the Class B and C nonspecific 5’, 3’-nucleotidase theme making available products required for nucleotide biosynthesis [32, 33]. Interestingly, this enzyme removed phosphate from both NADP^+^ and NADPH albeit to a lesser extent than that observed for other phosphorylated substrates, *i*.*e*., 3.7 and 3.5%, respectively. Could removal of the 2’-phosphate be prefatory to conversion of NAD^+^ to nicotinamide mononucleotide (NMN) and AMP, thus constituting a vestigial NAD^+^ utilization pathway [34, 35]? It remains to be determined if this phosphatase plays a role in the pathogenesis of this organism, the topic of ongoing investigation.

## Supporting information

S1 Raw images (PDF)
