# Supplementary figures and images for: Biochemical characterization of a recombinant acid phosphatase from Acinetobacter baumannii
Source: PLoS One. 2021 Jun 2;16(6):e0252377. doi: 10.1371/journal.pone.0252377 (PMC8172068; doi:10.1371/journal.pone.0252377)

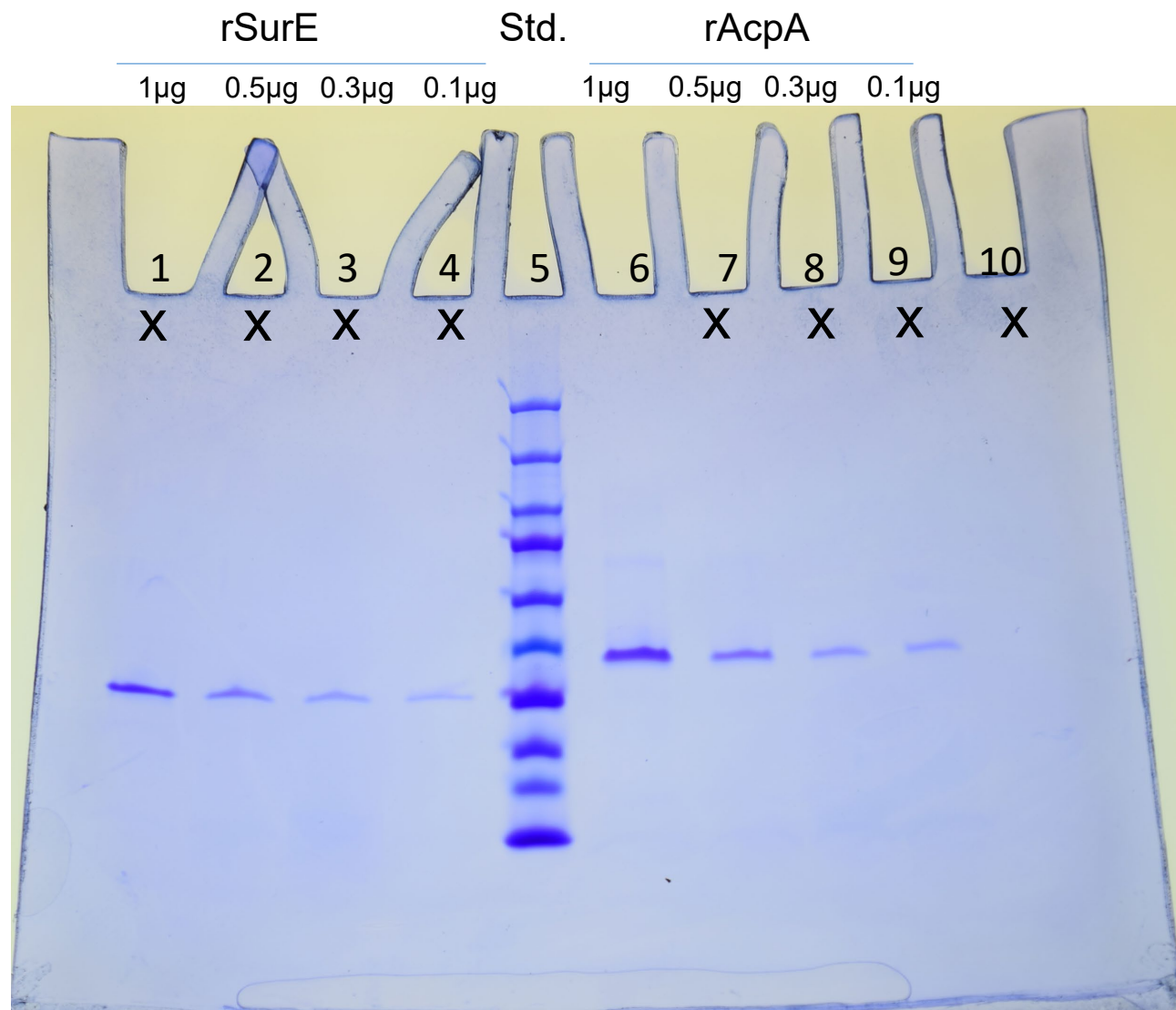

The image was taken with a Nikon camera.  
Lanes 5 and 6 were used in Fig 1A.

Supplement: S1 Raw images — (PDF) [file pone.0252377.s001.pdf]
